# Pharmacological Targeting of the Hepcidin/Ferroportin Axis

**DOI:** 10.3389/fphar.2016.00160

**Published:** 2016-06-21

**Authors:** Giada Sebastiani, Nicole Wilkinson, Kostas Pantopoulos

**Affiliations:** ^1^Department of Medicine, McGill UniversityMontreal, QC, Canada; ^2^Division of Gastroenterology, Royal Victoria HospitalMontreal, QC, Canada; ^3^Lady Davis Institute for Medical Research, Jewish General HospitalMontreal, QC, Canada

**Keywords:** iron metabolism, hemochromatosis, anemia, inflammation, erythropoiesis

## Abstract

The iron regulatory hormone hepcidin limits iron fluxes to the bloodstream by promoting degradation of the iron exporter ferroportin in target cells. Hepcidin insufficiency causes hyperabsorption of dietary iron, hyperferremia and tissue iron overload, which are hallmarks of hereditary hemochromatosis. Similar responses are also observed in iron-loading anemias due to ineffective erythropoiesis (such as thalassemias, dyserythropoietic anemias and myelodysplastic syndromes) and in chronic liver diseases. On the other hand, excessive hepcidin expression inhibits dietary iron absorption and leads to hypoferremia and iron retention within tissue macrophages. This reduces iron availability for erythroblasts and contributes to the development of anemias with iron-restricted erythropoiesis (such as anemia of chronic disease and iron-refractory iron-deficiency anemia). Pharmacological targeting of the hepcidin/ferroportin axis may offer considerable therapeutic benefits by correcting iron traffic. This review summarizes the principles underlying the development of hepcidin-based therapies for the treatment of iron-related disorders, and discusses the emerging strategies for manipulating hepcidin pathways.

## Introduction

**Figure 1:**
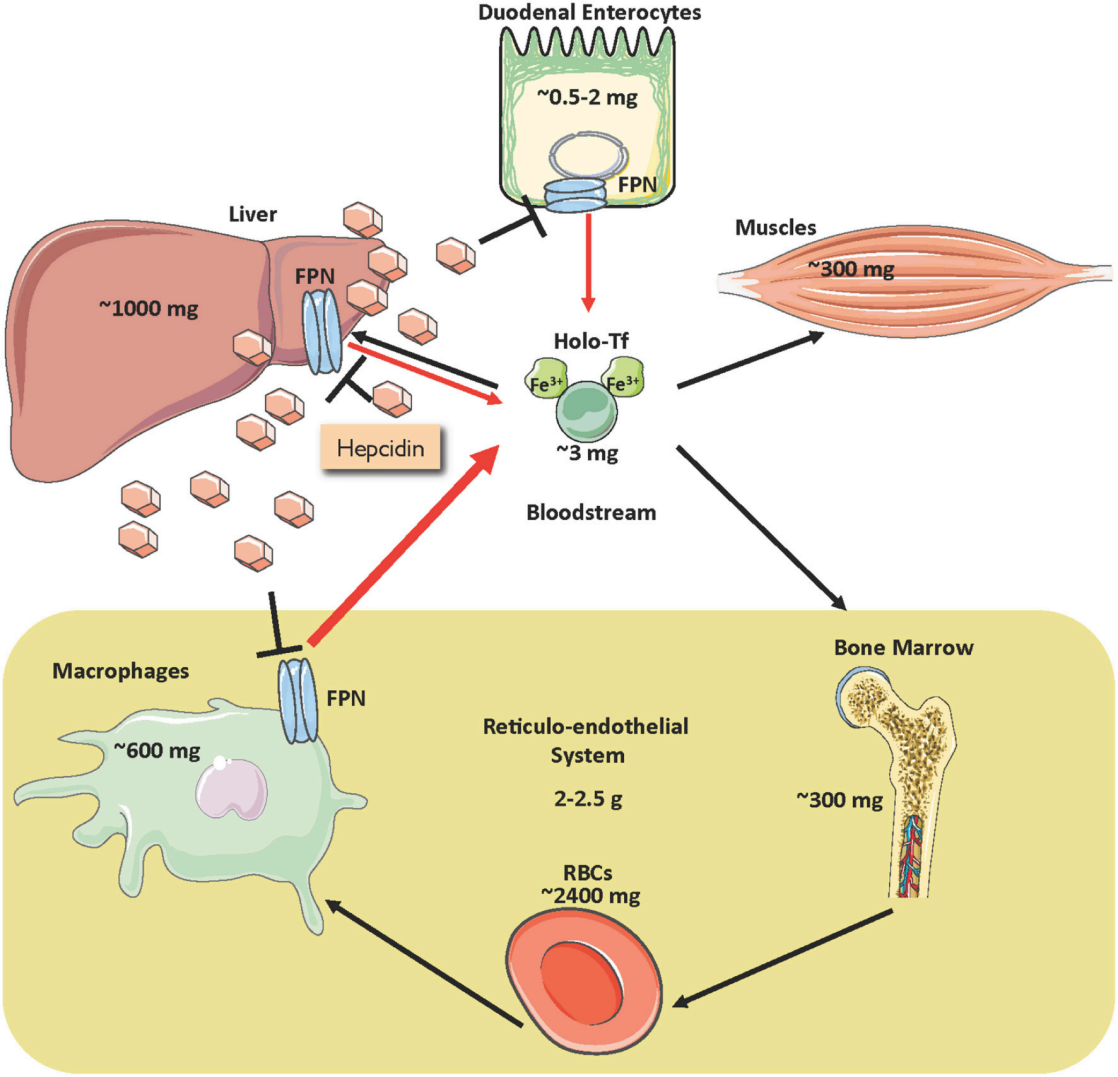
**Distribution of iron in the adult human body and regulation of iron traffic**. Circulating iron is bound to transferrin (holo-Tf) and delivered to tissues (black arrows). Holo-Tf is primarily replenished by iron recycled from tissue macrophages (thick red arrow), but also by dietary iron absorbed by duodenal enterocytes (thin red arrow). Under conditions of iron deficiency, iron stored in hepatocytes can also be mobilized (thin red arrow). Iron efflux to the bloodstream is inhibited by the liver-derived peptide hormone hepcidin, which binds to the iron exporter ferroportin (FPN) and promotes its degradation.

Iron is an essential constituent of hemoglobin, myoglobin and several other proteins, but also potentially toxic due to its redox reactivity that promotes oxidative stress (Papanikolaou and Pantopoulos, 2005). Balanced iron homeostasis is required to satisfy metabolic needs and minimize the risk of toxic side effects. More than two thirds of body iron (3–5 g in adults) is used in hemoglobin of red blood cells (RBCs) (Gkouvatsos et al., 2012). Erythroblasts require a daily supply of ~20–30 mg and non-erythroid cells another ~5 mg of iron, which is provided by plasma transferrin (Figure 1). The transferrin iron pool does not exceed ~3 mg at steady state and turns over >10 times/day. It is mostly replenished with iron recycled from senescent RBCs by tissue macrophages. The contribution of dietary iron absorption (1–2 mg/day) is minimal in healthy individuals, and mostly compensates for non-specific iron losses due to cell desquamation or bleeding. Iron stored in hepatocytes can be mobilized for erythropoiesis under conditions of iron deficiency.

Iron efflux to the bloodstream is physiologically restrained by hepcidin, an antimicrobial peptide and master hormonal regulator of systemic iron metabolism (Ganz, 2013). Hepcidin is synthesized in hepatocytes and secreted to the bloodstream for binding to the iron exporter ferroportin (FPN) in target cells, primarily macrophages and enterocytes, and to some extent hepatocytes (Figure 1). The binding of hepcidin promotes internalization and lysosomal degradation of ferroportin (Drakesmith et al., 2015). As a result, recycled iron remains in macrophages, while dietary iron absorption through the intestine is inhibited.

**Figure 2:**
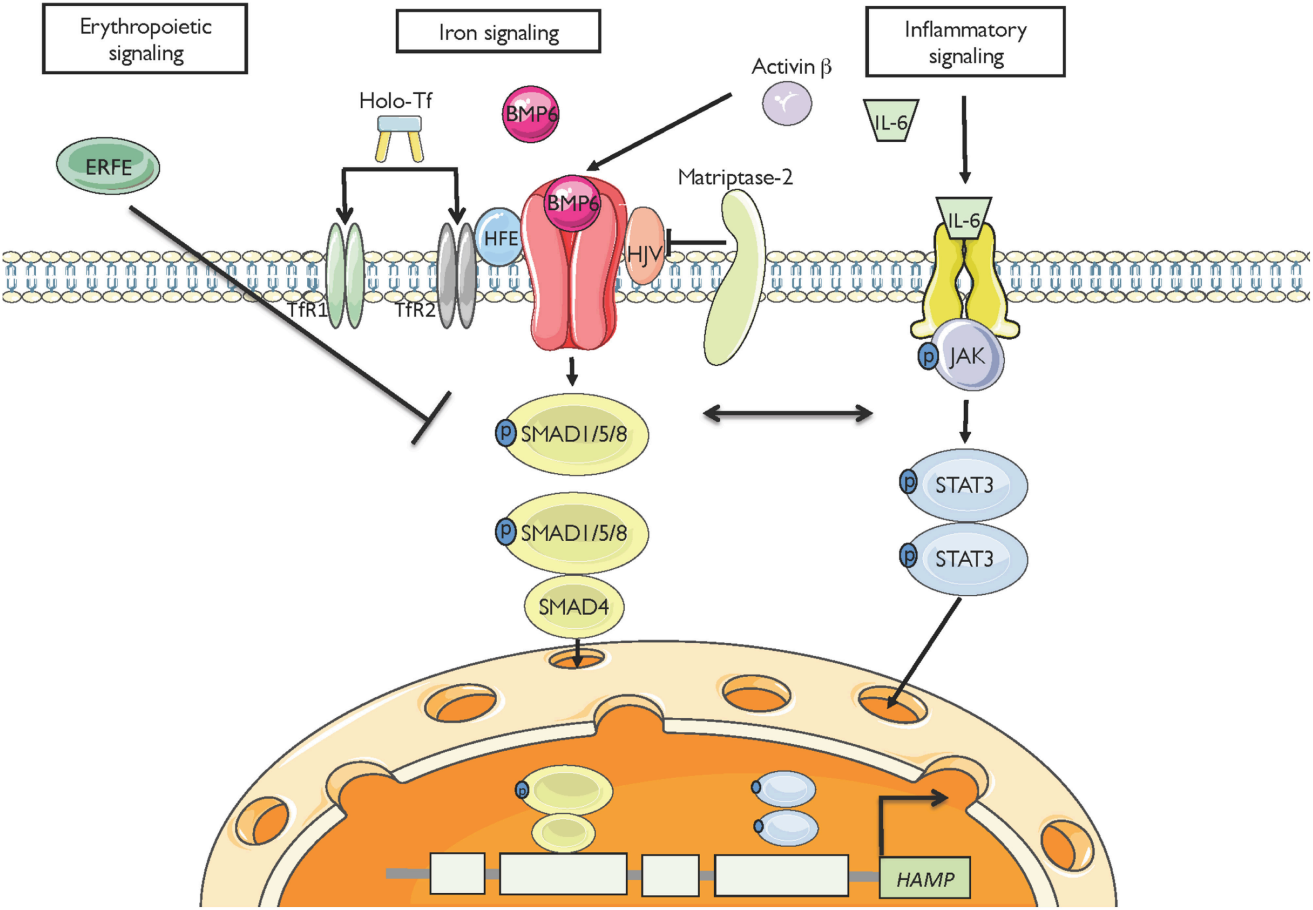
**Major pathways for hepcidin regulation**. High serum iron levels or hepatic iron stores (reflected in BMP6) induce hepcidin mRNA transcription via the BMP/SMAD signaling cascade. The inflammatory cytokines IL-6 and activin B induce hepcidin mRNA transcription via JAK/STAT and non-canonical BMP/SMAD signaling, respectively. High erythropoietic drive (reflected in ERFE) suppresses hepcidin transcription, likely via interference with BMP/SMAD signaling.

**Figure 3:**
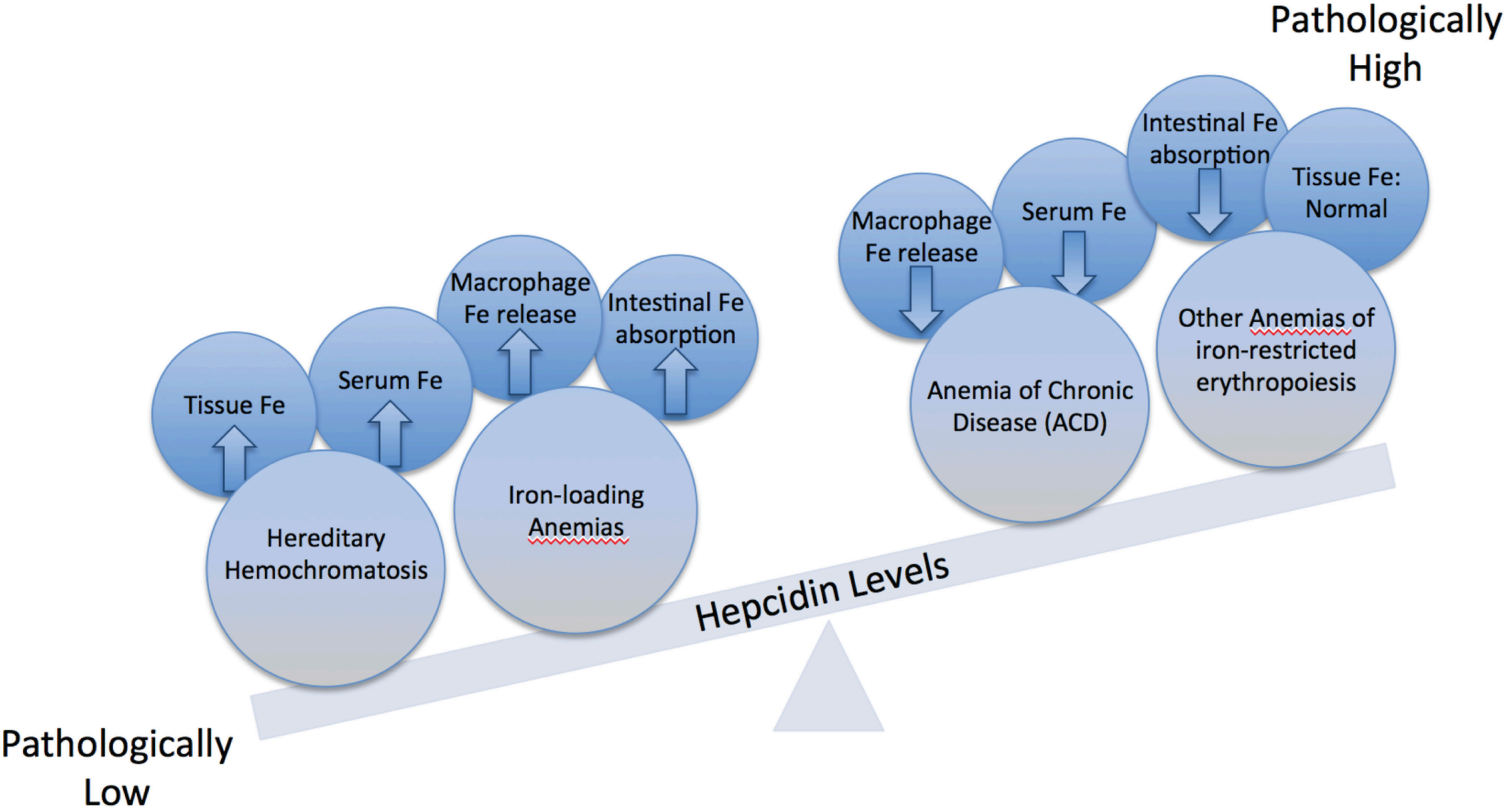
**Imbalance in hepcidin expression**. Physiological hepcidin responses correlate with healthy body iron metabolism. Pathologically low hepcidin expression occurs in hereditary hemochromatosis (HH) and in iron-loading anemias; this leads to hyperferremia and parenchymal tissue iron overload due to increased iron efflux to the bloodstream from macrophages and intestinal enterocytes. Pathologically high hepcidin expression occurs in the anemia of chronic disease (ACD) and other anemias of iron-restricted erythropoiesis; this leads to hypoferremia and decreased iron availability for erythropoiesis due to iron sequestration in macrophages.

Hepcidin expression is predominantly modulated by iron, inflammation and erythropoiesis (Figure 2), but also responds to other stimuli such as endoplasmic reticulum stress, oxidative stress, gluconeogenesis, gonadal hormones, growth factors and hypoxia (Ganz and Nemeth, 2015; Kim and Nemeth, 2015; Pietrangelo, 2016; Wang and Babitt, 2016). Increased serum or tissue iron levels (reflected in transferrin saturation or expression of hepatic BMP6, respectively) promote hepcidin induction via BMP/SMAD signaling. Inflammatory IL-6 triggers hepcidin induction via IL-6/STAT signaling, in crosstalk with the BMP/SMAD pathway. Activin B is another inflammatory cytokine that activates hepcidin via non-canonical BMP/SMAD signaling. Increased erythropoietic activity orchestrated by erythropoietin leads to hepcidin suppression via erythroferrone (ERFE) and other cytokines. It is not well understood how ERFE suppresses hepcidin, but it appears to attenuate BMP/SMAD signaling (Nai et al., 2016). Misregulation of hepcidin is etiologically linked or contributes to iron-related disorders (listed in Table 1), in which pharmacological targeting of the hepcidin/ferroportin axis to restore physiological hepcidin levels is expected to offer therapeutic benefits (Poli et al., 2014c; Ruchala and Nemeth, 2014; Schmidt and Fleming, 2014; Rochette et al., 2015; Blanchette et al., 2016; Liu et al., 2016). Pathological implications of hepcidin misregulation are schematically outlined in Figure 3 and discussed below.

**Table 1:** **Disorders associated with misregulation of hepcidin**.

**Disorders with hepcidin deficiency**
Hereditary hemochromatosis (genetic suppression of hepcidin)
adult forms caused by mutations in *HFE* or *TFR2*
juvenile forms caused by mutations in *HJV* or *HAMP*
Iron-loading anemias (erythropoietic suppression of hepcidin)
thalassemias
dyserythropoietic anemias
myelodysplastic syndromes
Chronic liver diseases (suppression of hepcidin by oxidative stress)
chronic hepatitis C
**Disorders with hepcidin excess**
Anemia of chronic disease (inflammatory induction of hepcidin)
observed in various chronic inflammatory conditions and some cancers
Other anemias with iron-restricted erythropoiesis
Anemia of chronic kidney disease (inflammatory induction and reduced renal
clearance of hepcidin)
Iron-refractory iron deficiency anemia (genetic induction of hepcidin caused
by mutations in *TMPRSS6*)
Anemia of Castleman disease (inflammatory induction of hepcidin triggered
by tumor-derived IL-6

## Disorders of hepcidin deficiency

Hereditary hemochromatosis (HH) is an endocrine disorder of systemic iron overload that is caused by hepcidin insufficiency (Pietrangelo, 2015; Powell et al., 2016). It constitutes the most frequent genetic disorder in populations of Northern European ancestry and is characterized by chronic hyperabsorption of dietary iron (up to 8–10 mg/day) and unrestricted release of iron from macrophages. These responses lead to hyperferremia, gradual saturation of transferrin and buildup of redox-active non-transferrin bound iron (NTBI), which is deposited within tissue parenchymal cells. HH patients fail to mount appropriate iron-dependent hepcidin induction due to mutations in upstream regulators of iron signaling to hepcidin (HFE, TfR2 or HJV; see Figure 2) or disruption of the hepcidin gene (*HAMP*). Patients with non-classical ferroportin disease, carrying mutations in ferroportin that prevent hepcidin binding, develop clinical features of HH due to hepcidin resistance (Mayr et al., 2010).

HH is genetically heterogeneous and its severity correlates with the degree of inhibition in hepcidin induction relative to body iron stores. The prevalent *HFE*-related variant exhibits a relatively milder phenotype due to residual hepcidin responsiveness. Rare variants (*TFR2*-, *HJV*- or *HAMP*-related) are associated with more profound iron overload and hepcidin inactivation. Disruption of either *HJV* or *HAMP* genes leads to early onset juvenile HH, the most severe form of the disease.

Clinical complications of adult HH develop after the fourth decade of life and include liver pathology (fibrosis, cirrhosis, hepatocellular cancer), diabetes, skin hyperpigmentation, arthritis and osteoporosis. Juvenile HH patients usually present with hypogonadism in their early 20s and develop fatal cardiomyopathy before the age of 30 if untreated. The standard of care for all forms of HH is reduction of iron burden via therapeutic phlebotomy (Sivakumar and Powell, 2016). This approach is effective and cheap. However, some patients are intolerant, or have low acceptance and compliance to a life-long treatment, or present contraindications (severe heart disease or anemia). These patients are good candidates for new therapies based on restoration of appropriate hepcidin levels.

Hepcidin deficiency is common in hematological disorders associated with ineffective erythropoiesis, such as thalassemias, dyserythropoietic anemias and myelodysplastic syndromes, and contributes to iron overload (Ginzburg and Rivella, 2011; Camaschella and Nai, 2016). Ineffective erythropoiesis is characterized by massive expansion of bone marrow erythroblasts due to decreased production of mature RBCs. This creates a high demand for iron, which leads to suppression of hepcidin in spite of systemic iron overload. Evidently, the negative erythropoietic signals dominate over the positive iron signals under these conditions. Erythropoietic suppression of hepcidin triggers iron overload in non-transfused patients with mild forms of iron loading anemias, and aggravates secondary iron overload in transfused patients (Ginzburg and Rivella, 2011). Restoration of hepcidin could prevent iron overload in the former and improve the efficacy of iron chelation therapy in the latter.

Inhibition of hepcidin expression also contributes to iron overload observed in chronic hepatitis C and other chronic liver diseases (Sebastiani and Pantopoulos, 2011; Pietrangelo, 2016). This is mainly attributed to oxidative stress mechanisms, which appear to override hepcidin-inducing inflammatory signals. Eradication of hepatitis C virus with direct-acting antiviral (DAA) drugs (Zopf et al., 2016) is expected to restore hepcidin expression without need for further interventions.

## Disorders of hepcidin excess

Excessive hepcidin expression is commonly observed in chronic inflammatory conditions due to infectious or autoimmune disorders or cancer (Weiss, 2015; Wang and Babitt, 2016). Inflammatory induction of hepcidin is primarily mediated by IL-6 and leads to hypoferremia due to ferroportin degradation and iron sequestration in tissue macrophages. Remodeling of iron metabolism by hepcidin-independent mechanisms may further exacerbate this phenotype. Thus, lipopolysaccharide (LPS) and interferon-γ (IFN-γ) inhibit iron efflux from monocytes by decreasing ferroportin expression (Ludwiczek et al., 2003), while the Toll-like receptor 2 and 6 (TLR2/6) ligands FSL1 or PAM3CSK4 trigger hypoferemia in mice by suppressing ferroportin transcription in tissue macrophages (Guida et al., 2015).

The acute hypoferremic response is considered to be protective against infection by depriving bacteria from iron, and may also be enhanced by antimicrobial activities of hepcidin. However, persistent chronic hypoferremia restricts iron availability for erythropoiesis (Ganz and Nemeth, 2015). Together with immune-driven reduced proliferation and life span of RBCs, the diversion of iron traffic contributes to pathogenesis of the anemia of chronic disease (ACD), or anemia of inflammation, the most frequent anemia among hospitalized patients (Weiss, 2015). ACD is typically normocytic/normochromic and unassociated with a reduction in body iron stores, but may be confounded by true iron deficiency due to chronic blood losses and/or scarcity or malabsorption of dietary iron. ACD patients with true iron deficiency exhibit reduced hepcidin levels and a microcytic/hypochromic phenotype.

Correction of ACD improves quality of patients' life. The best strategy is the successful treatment of the primary underlying cause. When this is not possible, ACD is often managed with erythropoiesis-stimulating agents (ESAs), combined or not with oral or intravenous iron administration or RBC transfusions. Nevertheless, these approaches are not always efficacious because hepcidin overexpression blunts responses to ESAs and maintains iron unavailable to erythroblasts. Therefore, they could be complemented by strategies to lower hepcidin levels, thereby mitigating erythropoietic iron-restriction.

Patients with chronic kidney disease (CKD) accumulate high hepcidin levels in the bloodstream due to reduced renal clearance, but also due to inflammatory induction of hepcidin transcription (Tsuchiya and Nitta, 2013). This is associated with iron-restricted erythropoiesis and contributes to anemia. Moreover, it negatively affects therapy with ESAs and oral or intravenous iron. Thus, hepcidin-lowering strategies could improve therapeutic outcomes.

Genetic inactivation of the *TMPRSS6* gene leads to unrestricted hepcidin production in spite of low body iron stores, which underlies the pathogenesis of *iron-refractory iron deficiency anemia* (IRIDA) (Heeney and Finberg, 2014). *TMPRSS6* encodes matriptase-2, a transmembrane serine protease that negatively regulates BMP/SMAD signaling to hepcidin (see Figure 2). It appears that hepcidin overexpression is the sole driver of IRIDA, which is microcytic and hypochromic. High hepcidin levels render IRIDA patients unresponsive to oral and partially responsive to intravenous iron therapy. Here, correction of hepcidin excess would not only improve responsiveness to iron therapy, but also provide an etiologic cure.

Pediatric patients with Castleman disease develop a chronic inflammatory anemia with IRIDA-like features. Castleman disease is caused by tumors overproducing IL-6, which in turn promotes an inflammatory state and excessive hepcidin expression (Arlet et al., 2010). The ensuing anemia is refractory to oral iron therapy and can be reversed by resection of the tumor, which eliminates the hepcidin inducer (IL-6). Pharmacological reduction of hepcidin could offer another option for anemia management.

All above-described conditions constitute disorders of systemic hepcidin excess, where high circulating hepcidin levels are derived from hepatocytes. Nevertheless, different cell types in several tissues can also produce hepcidin locally, and this may have profound pathophysiological ramifications. For instance, hepcidin generated by breast or prostate cancer epithelial cells promotes iron retention via autocrine degradation of ferroportin, which in turn favors survival and growth (Pinnix et al., 2010; Tesfay et al., 2015). Therefore, targeted delivery of hepcidin antagonists to hepcidin-overexpressing tumors may sensitize them to anti-cancer therapies.

Hepcidin is also produced in response to inflammation or other signals by heart cardiomyocytes (Merle et al., 2007) and by brain astrocytes and microglia (Urrutia et al., 2013). Considering that the hepcidin/ferroportin axis is crucial for cardiac function (Lakhal-Littleton et al., 2015) and that inflammatory induction of hepcidin causes iron accumulation in nervous system cells (Urrutia et al., 2013), localized targeting of hepcidin excess in the heart or brain may be important in the context of cardiovascular or neurodegenerative disorders.

## Pharmacological restoration of hepcidin

The therapeutic potential of restoring hepcidin levels in iron overload states is highlighted by genetic studies in mice. Thus, transgenic expression of hepcidin prevented iron overload in Hfe^−∕−^ (Nicolas et al., 2003) and Hbb^th3∕+^ (Gardenghi et al., 2010) mice, models of HH and β-thalassemia intermedia, respectively. Likewise, genetic disruption of *Tmprss6* enhanced BMP/SMAD signaling, increased endogenous hepcidin expression and prevented iron overload in Hfe^−∕−^ (Finberg et al., 2011) and Hbb^th3∕+^ mice (Nai et al., 2012). Moreover, manipulation of hepcidin levels by these strategies also improved erythropoiesis in Hbb^th3∕+^ mice (Gardenghi et al., 2010; Nai et al., 2012). It should be noted that disruption of both *Tmprss6* alleles offered optimal correction of iron overload but also caused microcytic anemia in Hfe^−∕−^ mice (Finberg et al., 2011), suggesting that titration of hepcidin levels within a physiological window is imperative to prevent adverse effects of hepcidin excess.

The protective effects of *Tmprss6* ablation in the Hfe^−∕−^ and Hbb^th3∕+^ backgrounds rendered this gene a candidate pharmacological target for therapeutic hepcidin induction. In fact, suppression of hepatic *Tmprss6* by using RNAi (Schmidt et al., 2013) or antisense oligonucleotides (Guo et al., 2013a) increased hepcidin expression in wild type, Hfe^−∕−^ and Hbb^th3∕+^ mice. Importantly, it reduced systemic iron overload in Hfe^−∕−^ and Hbb^th3∕+^ mice, and ameliorated anemia in Hbb^th3∕+^ mice. The development of oligonucleotide therapeutics against target genes in the liver is an active area of research (Sehgal et al., 2013). Limitations are related to potential off-target effects and toxicity, pharmacokinetic problems of delivery and clearance from the bloodstream, as well as high cost.

Administration of recombinant BMP6 was shown to improve iron signaling to hepcidin and correct systemic iron homeostasis in Hfe^−∕−^ mice, even though endogenous BMP6 expression is appropriately induced in these animals (Corradini et al., 2010). Nevertheless, prolonged BMP6 application caused peritoneal calcifications, indicative of lack of target specificity, which may lead to further pleiotropic side effects.

Cell culture studies and chemical screens identified several small molecules capable of stimulating hepcidin transcription *in vitro*, such as genistein (Zhen et al., 2013), SB204741, daunorubicin, ethacridine, phenazopyridine, 9-aminoacridine, amlexanox, lansoprazole, leflunomide, ipriflavone, AS252424, pterostilbene, AG1296, GTP14564, SU6668, leflunomide, 10058-F4 and vorinostat (Gaun et al., 2014). An RNAi screen identified sorafenib, wortmannin, rapamycin and metformin as inducers of hepcidin mRNA expression (Mleczko-Sanecka et al., 2014). These drugs affect different pathways, including growth factor signaling, anti-inflammatory signaling, DNA repair and apoptosis. Their exact mechanisms of action are not well understood and their capacity to control hepcidin expression *in vivo* has not been examined. Broad target specificity may disqualify many of the above-described drugs for pharmacological applications to stimulate hepcidin.

Another small molecule screen identified three steroid molecules as hepcidin inducers (Li et al., 2016). Progesterone, epitiostanol, and mifepristone were shown to stimulate hepcidin transcription independently of the BMP/SMAD and IL-6/STAT3 pathways, by a mechanism requiring progesterone receptor membrane component-1 (PGRMC1). Administration of progesterone to women in the context of standard fertility treatments resulted in hepcidin induction (Li et al., 2016). Contrary to progesterone, 17β-estradiol inhibits hepcidin transcription via an estrogen-responsive promoter element (Yang et al., 2012). Testosterone likewise inhibits hepcidin but operates by enhancing negative epidermal growth factor receptor (EGFR) signaling (Latour et al., 2014), and by competing positive BMP signaling via androgen receptor binding to SMAD proteins (Guo et al., 2013b). Hepcidin-inducing steroids or drugs that lower 17β-estradiol or testosterone could be applied for pharmacological restoration of hepcidin. Nevertheless, potential long-term side effects of steroids should be considered.

**Figure 4:**
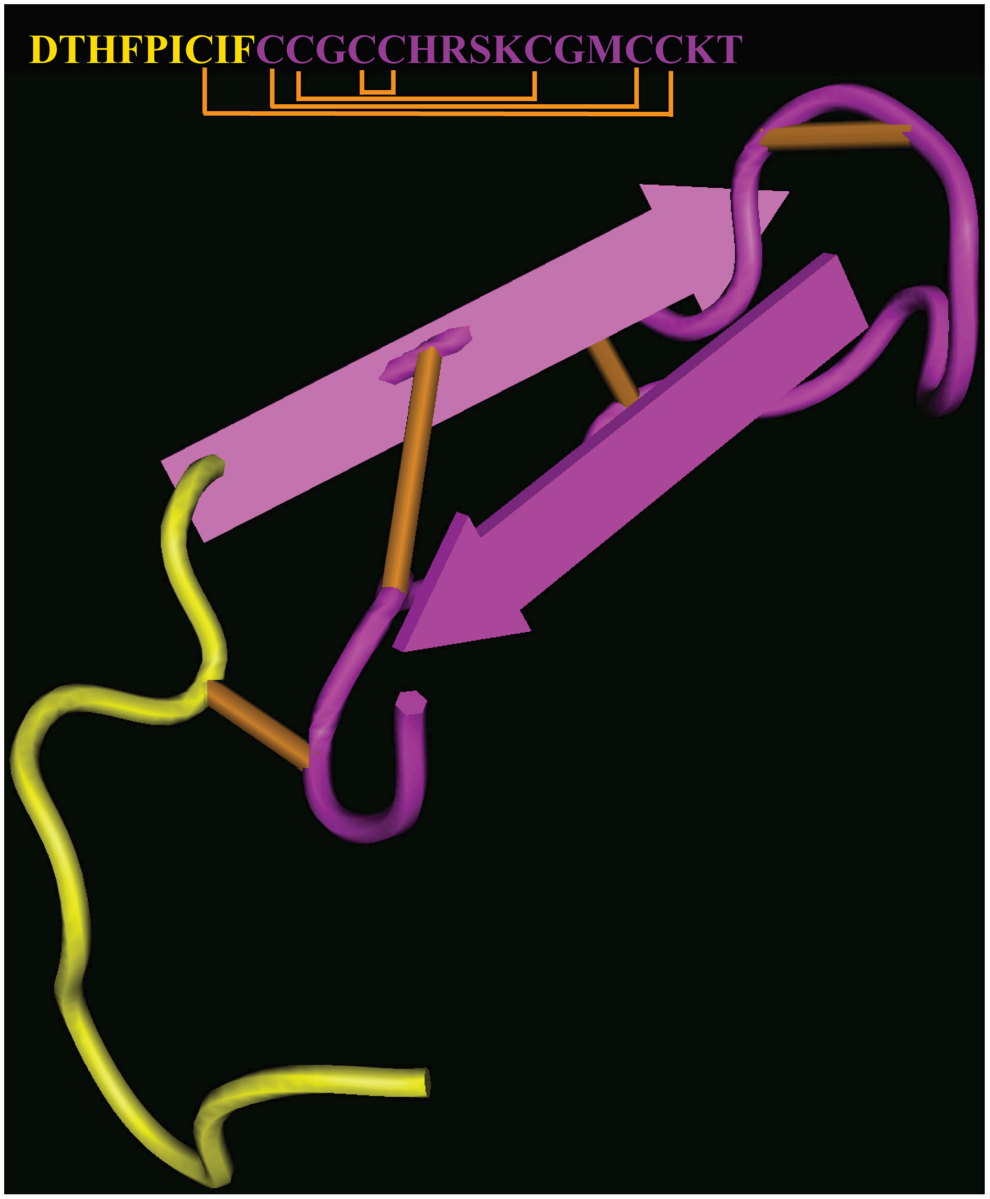
**Crystal structure and sequence of human hepcidin**. The nine N-terminal amino acids involved in binding to ferroportin are highlighted in yellow (PDB ID 1M4F). Disulfide bonds are highlighted in orange.

Hepcidin supplementation therapy would provide a straightforward approach, devoid of inherent limitations associated with the targeting of upstream regulators. However, the chemical synthesis of hepcidin or its expression as recombinant peptide is costly, while appropriate folding to the biologically active form is complicated by the presence of 8 cysteine residues within the 25 amino acids of the mature peptide forming 4 disulfide bridges (Figure 4). Moreover, synthetic or recombinant hepcidin is rapidly cleared in the circulation and therefore pharmacological concentrations cannot be sustained. Encapsulation of hepcidin into biocompatible nanocarriers suitable for controlled release of therapeutics (Cheng et al., 2015) could theoretically address this issue. A formulation of hepcidin developed by La Jolla Pharmaceutical Company (LJPC-401) is currently undergoing Phase 1 clinical trials with patients at risk of iron overload (http://lajollapharmaceutical.com/2015/10/la-jolla-pharmaceutical-company-doses-first-patient-in-phase-1-clinical-trial-of-ljpc-401-in-patients-at-risk-of-iron-overload/).

Biochemical studies revealed that hepcidin interacts with ferroportin via its N-terminal region (highlighted in Figure 4), and that C-terminal amino acid residues are dispensable. This led to the development of mini-hepcidin analogs containing a minimal core of 7–9 N-terminal amino acids with a single cysteine, which suffice to trigger ferroportin internalization and degradation in cells and mice (Preza et al., 2011). Substitution of natural with modified amino acids and introduction of polyethylene glycol (PEG) and hydrophobic linkers (palmitic acid) improved stability and pharmacological properties of mini-hepcidins. Cyclization may increase stability but appears to reduce biological activity (Chua et al., 2015). Treatment with an engineered mini-hepcidin mimetic (PR65) prevented iron overload in iron-depleted hepcidin-deficient Hamp^−∕−^ mice, but was less efficient in correcting iron traffic when these mice were already iron overloaded (Ramos et al., 2012). High doses of PR65 caused anemia, emphasizing the importance of titration. The mini-hepcidin pro-drug M009 is metabolized to active mini-hepcidin M004 and was recently shown to improve erythropoiesis in Hbb^th3∕+^ mice and in a mouse model of polycythemia vera (Casu et al., 2016). Another mini-hepcidin derivative developed by Merganser Biotech (M012) initiated a clinical trial in February 2016 (http://merganserbiotech.com/2016/02/24/merganser-biotech-inc-initiates-first-clinical-trial/).

Interestingly, administration of the mini-hepcidin mimetic PR73 protected Hamp^−∕−^ mice against infection with the siderophilic bacterium *Vibrio vulnificus* (Arezes et al., 2015). PR73 caused hypoferremia to these animals in spite of tissue iron overload, which restricted circulating iron from *V. vulnificus* and thereby inhibited its growth. Considering that infections with extracellular siderophilic pathogens can be lethal to HH patients (Frank et al., 2011), these data highlight another possible application of hepcidin agonists. Finally, the adverse effects of hepcidin inactivation in mouse models of malaria infection (Portugal et al., 2011) or microbial sepsis suggest a therapeutic potential of hepcidin agonists in the context of these pathologies (Zeng et al., 2015).

## Pharmacological reduction of hepcidin

Targeting of hepcidin pathways is expected to correct defects triggered by excessive hepcidin expression. Supporting evidence is provided by studies with multicentric Castleman disease patients who were treated with therapeutic monoclonal antibodies against IL-6 receptor (tocilizumab) or IL-6 (siltuximab). These treatments reduced hepcidin levels and improved anemia (Song et al., 2010; Casper et al., 2015). Tocilizumab administration also lowered hepcidin and improved anemia in rheumatoid arthritis patients (Isaacs et al., 2013; Song et al., 2013) and in a monkey arthritis model (Hashizume et al., 2010). Similar results were obtained when rheumatoid arthritis patients were treated with antibodies against TNFα (golimumab or infliximab), possibly as an indirect result of concomitant suppression of IL-6 (Doyle et al., 2013; Song et al., 2013).

Studies with cell and mouse models showed that downstream inhibition of the IL-6/STAT3 signaling pathway by using small molecule inhibitors of STAT3 (curcumin, AG490 and PpYLKTK) likewise decreases hepcidin (Jiao et al., 2009; Fatih et al., 2010; Zhang et al., 2011). However, further development of this class of drugs as hepcidin inhibitors is hindered by lack of specificity (all STAT3 inhibitors), competing iron chelating properties (curcumin) or poor pharmacokinetics (AG490 and PpYLKTK).

As the IL-6/STAT3 and BMP/SMAD signaling pathways are tightly connected (Wang and Babitt, 2016), targeting the latter has the potential to efficiently antagonize hepcidin induction under inflammatory conditions. One strategy exploited the high affinity of BMPs to heparin, a glycosaminoglycan that is clinically applied as anticoagulant. Thus, heparin was shown to inhibit hepcidin expression in cells and mice by sequestering BMPs, while patients treated with heparin for prevention of deep vein thrombosis had reduced serum hepcidin levels (Poli et al., 2011). Because the anticoagulant properties of heparin are undesired for interventions to reduce hepcidin excess, glycol-split non-anticoagulant heparins were tested and found to be equally effective as hepcidin inhibitors; moreover they improved anemia in mouse models of inflammation (Poli et al., 2014a). Highly sulfated heparins with low anticoagulant activity likewise inhibited hepcidin in cells and mice (Poli et al., 2014b). Preservation of high sulfation grade and molecular weight are critical for their hepcidin inhibitory activity (Asperti et al., 2016). Glycol-split and highly sulfated heparins are good candidates for pharmacological targeting of hepcidin in clinical settings and are amenable to further development. Their efficacy and safety profile needs to be established in randomized controlled trials.

HJV is a BMP co-receptor that enhances iron signaling to hepcidin (Babitt et al., 2006). Its neutralization by using humanized monoclonal HJV antibodies (developed by Abbvie) reduced hepcidin expression in rats and cynomolgus monkeys (Böser et al., 2015). Moreover, they corrected anemia via hepcidin inhibition in rat and mouse models of ACD, and in Tmprss6^−∕−^ mice (Kovac et al., 2016). A secreted soluble HJV form (sHJV) functions as a competitive inhibitor of BMP binding to BMP receptors (Lin et al., 2005). Administration of sHJV.Fc, a fusion of sHJV with the Fc fragment of immunoglobulin G, reduced hepcidin and ameliorated anemia in a rat model of ACD (Theurl et al., 2011). Two clinical trials sponsored by Ferrumax Pharmaceuticals Inc aimed to use sHJV.Fc (FMX-8) for the treatment of patients with renal disease, but were not completed due to inability to recruit patients meeting eligibility criteria (ClinicalTrials.gov Identifiers: NCT01873534 and NCT02228655).

Preliminary data of hepcidin inhibition by targeting upstream regulators of the BMP/SMAD and IL-6/STAT3 pathways with oligonucleotide therapeutics were reported a few years ago (Akinc et al., 2011), but the current stage of development of these technologies is unknown.

Small molecule inhibitors of the BMP/SMAD pathway have been tested and shown to act as hepcidin-lowering agents. Thus, dorsomorphin, an inhibitor of the type I BMP receptors ALK2, ALK3, and ALK6, prevented hepcidin induction by IL-6 in cells and induced hyperferremia due to hepcidin suppression in mice (Yu et al., 2008). Furthermore, the dorsomorphin derivative LDN-193189, antagonized inflammatory induction of hepcidin in cells and mitigated anemia in rat (Theurl et al., 2011) and mouse (Steinbicker et al., 2011; Mayeur et al., 2015) models of ACD, and in a rat model of kidney disease (Sun et al., 2013). Interestingly, suppression of hepcidin by LDN-193189 was associated with anti-atherogenic responses in apoE^−∕−^ mice, which were reversed by exogenous hepcidin (Saeed et al., 2012). These findings suggest that pharmacological targeting of hepcidin may be valuable beyond the context of improving erythropoiesis. Nevertheless, dorsomorphin and LDN-193189 are unlikely to be considered for clinical application due to lack of target specificity (Boergermann et al., 2010). TP-0184, a specific ALK2 inhibitor developed by Tolero Pharmaceuticals, was recently shown to block inflammatory induction of hepcidin in mouse models of ACD and cancer-induced anemia (Peterson et al., 2015). Further studies are required to assess its suitability for clinical applications.

Anemia in elderly persons with chronic inflammatory conditions (Perlstein et al., 2011) and in CKD patients (Zughaier et al., 2014), is often associated with vitamin D deficiency. Biochemical studies showed that binding of 1,25-dihydroxy-vitamin D to its receptor directly suppresses hepcidin transcription by binding to a promoter element (Bacchetta et al., 2014). Importantly, pilot clinical studies demonstrated that vitamin D supplementation can decrease hepcidin levels in healthy volunteers (Bacchetta et al., 2014) and early stage CKD patients (Zughaier et al., 2014). These findings underline the potential pharmacological value of vitamin D as a hepcidin-lowering agent, which needs to be further explored in animal models and randomized controlled trials.

Direct inhibition of hepcidin by neutralizing antibodies or other inhibitory molecules offers further opportunities for mitigating the adverse effects of hepcidin overexpression. Neutralizing hepcidin antibodies were shown to modulate iron metabolism in mice and cynomolgus monkeys; moreover, they improved erythropoiesis and responses to ESA therapy in mouse models of ACD (Sasu et al., 2010; Cooke et al., 2013). LY2787106, a monoclonal hepcidin antibody developed by Eli Lily and Company was evaluated in a Phase 1 clinical trial in patients with cancer-associated anemia (ClinicalTrials.gov Identifier: NCT01340976). The antibody treatment was well tolerated and resulted in transient iron mobilization and increased reticulocyte count relative to baseline (Vadhan-Raj et al., 2015).

Neutralization of hepcidin is also possible by using relatively high molecular weight antagonists. Pieris Pharmaceuticals Inc generated PRS-080, a PEGylated anticalin protein that specifically binds to hepcidin and inhibits its activity. Following successful initial testing *in vitro* and in mice (Hohlbaum et al., 2011), the pharmacokinetic properties and safety profile of PRS-080 were assessed in a clinical trial with healthy volunteers (ClinicalTrials.gov Identifier: NCT02340572). The results were encouraging and a further trial is planned with anemic CKD patients (Moebius et al., 2015).

NOXXON Pharma AG generated NOX-H94 (Lexaptepid Pegol), a Spiegelmer hepcidin antagonist. This PEGylated non-natural mirror-image L-oligoribonucleotide binds with high affinity to hepcidin and operates as a specific inhibitor. NOX-H94 administration improved inflammation-related anemia in cynomolgus monkeys (Schwoebel et al., 2013). Moreover, NOX-H94 prevented hypoferremia in experimental human endotoxemia with LPS-injected volunteers (Van Eijk et al., 2014) (ClinicalTrials.gov Identifier: NCT01522794). NOX-H94 is currently being evaluated in another three Phase 1 and Phase 2 clinical trials on patients with cancer-related anemia, ACD or CKD (ClinicalTrials.gov Identifiers: NCT01691040, NCT01372137, and NCT02079896).

A chemical screen for small molecule hepcidin antagonists identified fursultiamine, a thiol-reactive thiamine derivative that binds to ferroportin on C326 and thereby precludes the binding of hepcidin (Fung et al., 2013). This drug is available over-the-counter for treating vitamin B1 deficiency. While it efficiently protected ferroportin against hepcidin in cultured cells, it failed to exhibit *in vivo* anti-hepcidin activity, possibly due to rapid plasma turnover. These results suggest that fursultiamine could only be further considered as a hepcidin-lowering agent if modified to stable derivatives, or delivered in formulations that enable sustained controlled release. Nevertheless, they also show that blocking the hepcidin-binding site of ferroportin offers another option for pharmacological targeting the hepcidin/ferroportin axis. Along these lines, Eli Lily and Company developed a monoclonal ferroportin antibody (LY2928057), which increased iron mobilization in cynomolgus monkeys by binding to ferroportin and thereby inhibiting access to hepcidin (Witcher et al., 2013). LY2928057 is currently being evaluated in two Phase 1 clinical trials with healthy volunteers and anemic CKD patients (ClinicalTrials.gov Identifiers: NCT01330953 and NCT01991483).

## Conclusions

Hepcidin is a master hormonal regulator of systemic iron homeostasis. Under physiological conditions, its expression remains within a relatively narrow window. Misregulation of hepcidin is associated with a broad spectrum of disorders ranging from iron overload states to anemias with iron-restricted erythropoiesis. Correction of hepcidin levels can provide an etiologic cure to some of these disorders (HH, IRIDA), or offer therapeutic benefits to others (thalassemia, ACD). Several agonists and antagonists of hepcidin have been developed. The ones that have been validated in preclinical or clinical settings are summarized in Table 2 (inducers/mimetics) and Table 3 (antagonists). These drugs act at different levels through the hepcidin/ferroportin axis. Promising candidates are currently being further evaluated in randomized controlled trials. A major challenge for using hepcidin therapeutics is to maintain physiological concentrations of circulating hepcidin, and thereby avoid a shift from hepcidin deficiency to excess and vice versa.

**Table 2:** **Hepcidin inducers and mimetics validated *in vivo***.

**Drug**	**Target**	**Reference**
recombinant BMP6	BMPRs	Corradini et al., 2010
RNAi	Tmprss6	Schmidt et al., 2013
antisense oligonucleotides	Tmprss6	Guo et al., 2013a
progesterone	PGRMC1	Li et al., 2016
LJPC-401 (hepcidin formulation)	FPN	La Jolla Pharmaceutical Company (http://www.lajollapharmaceutical.com)
PR65 (mini-hepcidin)	FPN	Ramos et al., 2012
M009 (pro-M004) M004 (mini-hepcidin)	FPN	Casu et al., 2016
M012 (mini-hepcidin)	FPN	Merganser Biotech (http://www.merganserbiotech.com)
PR73 (mini-hepcidin)	FPN	Arezes et al., 2015

**Table 3:** **Hepcidin antagonists validated *in vivo***.

**Drug**	**Target**	**Reference**
Tocilizumab (monoclonal IL-6 receptor Ab)	IL-6 receptor	Song et al., 2010
Siltuximab (monoclonal IL-6 Ab)	IL-6	Casper et al., 2015
Curcumin	STAT3	Jiao et al., 2009
AG490 (small molecule)	JAK2	Zhang et al., 2011
Heparin	BMPs	Poli et al., 2011
glycol-split heparins	BMPs	Poli et al., 2014a
highly sulfated heparins	BMPs	Poli et al., 2014b
monoclonal HJV Ab	HJV	Abbvie (www.abbvie.com)
sHJV.Fc	BMPs	Theurl et al., 2011
Dorsomorphin (small molecule)	type I BMPRs	Yu et al., 2008
LDN-193189 (small molecule)	type I BMPRs	Steinbicker et al., 2011
TP-0184 (small molecule)	ALK2	Tolero Pharmaceuticals (www.toleropharma.com)
1,25-dihydroxy-vitamin D	Vitamin D receptor	Bacchetta et al., 2014
17β–estradiol	estrogen-responsive promoter	Yang et al., 2012
Testosterone	EGFR	Latour et al., 2014
LY2787106 (monoclonal hepcidin Ab)	Hepcidin	Eli Lily and Company (www.lilly.com)
PRS-808 (PEGylated anticalin)	Hepcidin	Pieris Pharmaceuticals Inc (www.pieris.com)
NOX-H94 (PEGylated Spiegelmer)	Hepcidin	Schwoebel et al., 2013 NOXXON Pharma AG (www.noxxon.com)
LY2928057 (monoclonal FPN Ab)	FPN	Witcher et al., 2013 Eli Lily and Company (www.lilly.com)

## Author contributions

GS and NW contributed to writing the manuscript. KP wrote the manuscript.

### Conflict of interest statement

The authors declare that the research was conducted in the absence of any commercial or financial relationships that could be construed as a potential conflict of interest.
